# Granulomatosis with Polyangiitis Presenting as ANCA-Negative Pulmonary Disease with Distributive Shock

**DOI:** 10.3390/reports9020128

**Published:** 2026-04-21

**Authors:** Joel Shah, Emily Shah

**Affiliations:** 1 Department of Internal Medicine, Texas Tech University Health Sciences Center, 5001, El Paso, TX 79905, USA; 2 Department of Internal Medicine, William Beaumont Army Medical Center, El Paso, TX 79906, USA

**Keywords:** granulomatosis with polyangiitis, ANCA-negative, vasculitis, pulmonary nodules, distributive shock, case report

## Abstract

**Background:** Granulomatosis with polyangiitis (GPA) is an antineutrophil cytoplasmic antibody (ANCA)-associated necrotizing vasculitis primarily affecting small and medium-sized vessels. The typical presentation commonly includes upper and/or lower respiratory tract and renal involvement. GPA has a particularly strong association with proteinase-3 (PR3) ANCA. Though well defined, GPA may be clinically difficult to recognize, particularly in early disease. Initial presentations may include nonspecific symptoms, including but not limited to fatigue, fever, and sinus congestion or sinusitis, which may be mistaken for infection. Though initial ANCA testing is useful, it is not definitive as early stages of disease may be negative, thus delaying diagnosis; **Clinical Significance:** This case highlights the importance of including GPA in the differential diagnosis of patients with unremitting upper or lower respiratory and constitutional symptoms despite negative ANCA testing. Though atypical, GPA cases may lack renal involvement and even have negative ANCA serologies, leading to a delay in diagnosis and increased morbidity. ANCA positivity can be as low as 60% in limited GPA cases, and less than 20% of individuals have renal involvement at presentation. If GPA suspicion is high, repeat testing and biopsy are warranted; **Case Presentation:** A woman in her 50s initially presented to the emergency department with recurrent/persistent fever with nonspecific sinus symptoms that remained unresolved despite multiple outpatient treatments and tests. Infectious work-up was negative. She was found to have multiple pulmonary nodules on various scans. Initial testing on admission was unremarkable or nondiagnostic, including anti-neutrophil cytoplasmic antibody (ANCA) serologies. The patient’s hospital course was complicated by acute hypoxic respiratory failure with distributive shock during bronchoscopy. Repeat serological testing was positive for PR3-ANCA, and lung biopsy demonstrated necrotizing granulomatous vasculitis consistent with a diagnosis of granulomatosis with polyangiitis (GPA). The patient demonstrated clinical improvement with avacopan, glucocorticoids, and rituximab; **Conclusions:** The diagnosis of GPA should be suspected in all patients with nonspecific constitutional symptoms along with clinical evidence of upper/lower respiratory tract involvement, regardless of renal function. Physicians with a strong suspicion of an autoimmune disease, such as GPA, should utilize a thorough clinical history, physical exam, and other labs in the setting of a negative autoimmune marker and/or negative imaging. Clinical judgment is required to not rule out GPA despite a negative workup when other more serious causes have been excluded, as the diagnosis may be life-threatening.

## 1. Introduction

Granulomatosis with polyangiitis (GPA) is an antineutrophil cytoplasmic antibody (ANCA)-associated necrotizing vasculitis primarily affecting small and medium-sized vessels [1]. The typical presentation commonly includes upper and/or lower respiratory tract and renal involvement. GPA has a particularly strong association with proteinase-3 (PR3) ANCA. Though well defined, GPA may be clinically difficult to recognize, particularly in early disease. Initial presentations may include nonspecific symptoms, including but not limited to fatigue, fever, and sinus congestion or sinusitis, which may be mistaken for infection [2]. Though initial ANCA testing is useful, it is not definitive as early stages of disease may be negative, thus delaying diagnosis [3]. The term limited GPA refers to the development of disease without evidence of significant, life-threatening organ such as glomerulonephritis. It should be noted, however, that the term “limited” does not necessarily entail mild disease, and clinical deterioration may be present.

## 2. Case Presentation

**Figure 1:**
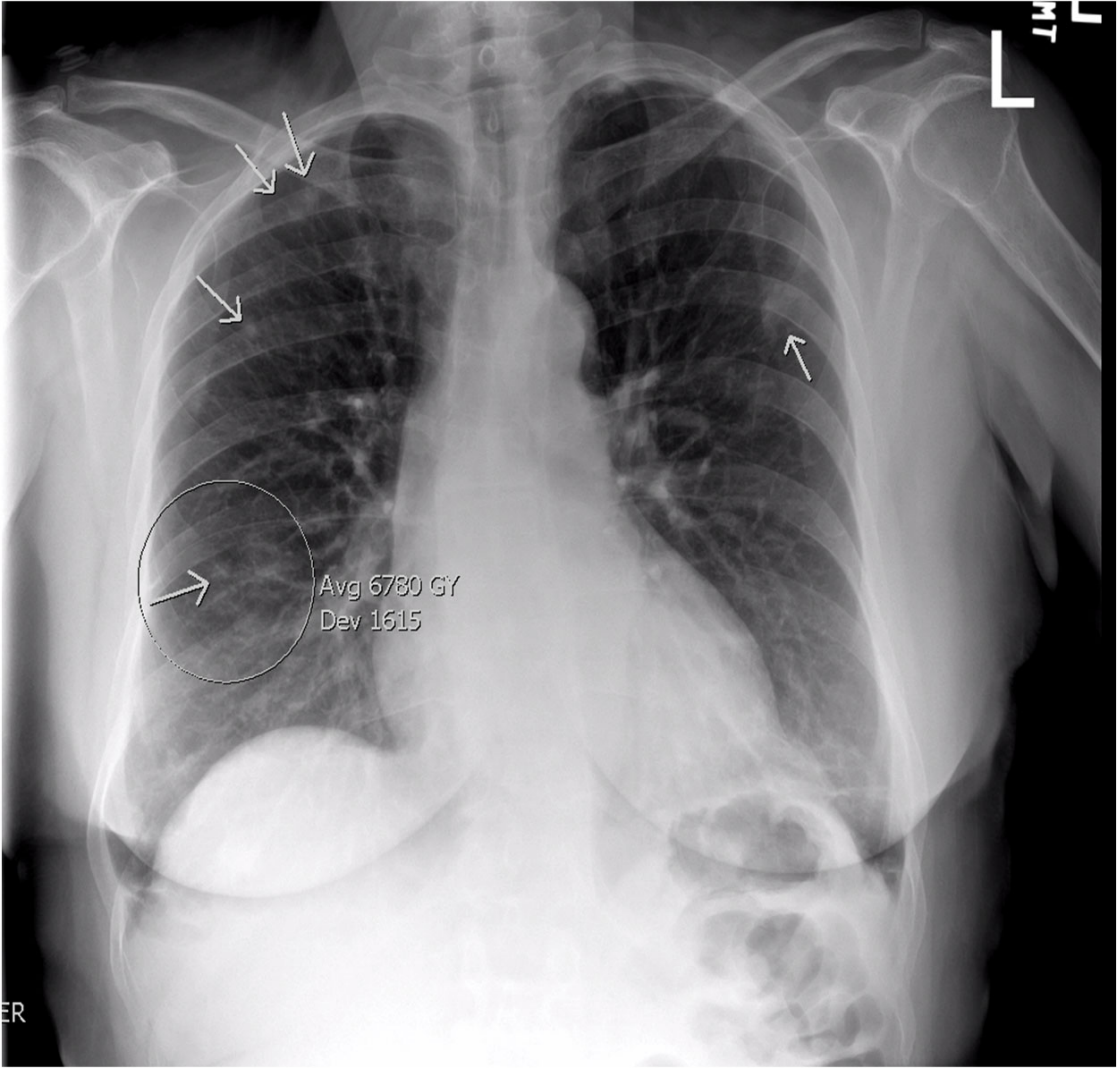
Chest X-ray revealing four nodules in the right lung and one in the left lung (white arrows).

**Figure 2:**
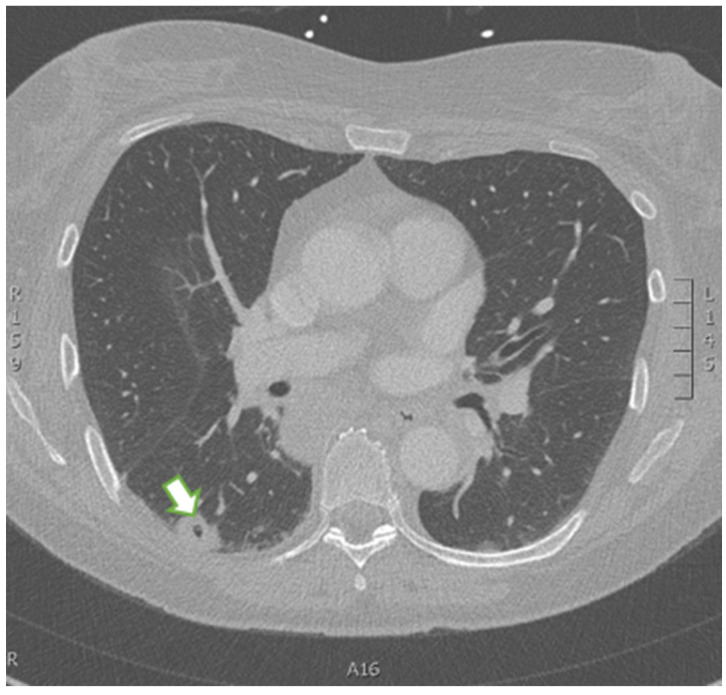
CT of the chest. The right lower lobe reveals evidence of a cavitary lesion (white arrow).

**Figure 3:**
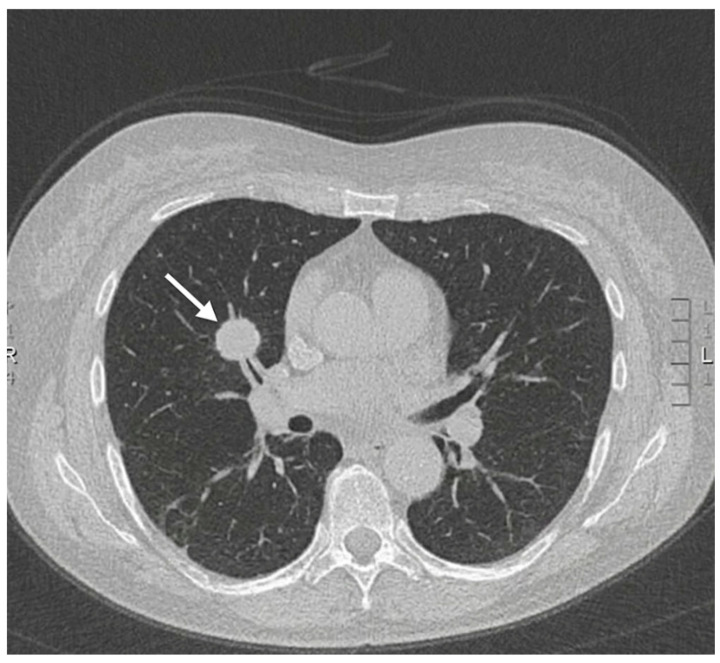
CT of the chest. A pulmonary nodule is noted in the right middle lobe (white arrow).

A 59-year-old female with a history of iron deficiency anemia presented to the emergency department (ED) with worsening bilateral maxillary sinus pain, headache, and fevers of one month’s duration. One month prior, she developed rhinorrhea and pain in her frontal and maxillary sinuses without fever. At that time, she was diagnosed with sinusitis by her primary care physician (PCP) and given a five-day course of azithromycin, which did not relieve her symptoms. One week later, the patient went to an ED due to her worsening sinus pain, where she was prescribed a 10-day course of amoxicillin/clavulanic acid and loratadine without improvement. She subsequently went to another ED three days later due to her worsening symptoms, where she was given a 5-day course of prednisone and fluticasone. She states her sinus-related symptoms resolved with steroid use, but she developed a fever of 38.8 C after completion of this medication course. Due to her unremitting fever and the development of a headache, she presented to a third ED, where she underwent CT imaging of the sinuses, which was interpreted as unremarkable. An additional CT of the chest was ordered due to the patient’s upper respiratory symptoms without a known etiology. At that time, autoimmune labs, including ANA with titers and patterns, as well as ANCA with Myeloperoxidase (MPO) and PR3 titers, were negative. The patient was subsequently referred to and evaluated by an otolaryngologist; however, her symptoms continued to worsen, and she developed a dry cough and fatigue alongside her fever and sinus pain. She returned to the ED, where a chest X-ray revealed the presence of bilateral lung nodules and a solitary cavity at the base of the right lung (Figure 1). CT of the chest and abdomen/pelvis confirmed the presence of a cavitary lesion in the right lower lobe of the lung (Figure 2), right hilar adenopathy, and multiple bilateral pulmonary nodules (Figure 3) concerning for malignancy and septic pulmonary emboli. CT of the neck, performed due to suspicions of Lemierre’s syndrome, given suspicions for pharyngitis, revealed no evidence of disease.

The patient was admitted to the internal medicine service for an expedited workup of her symptoms and lung nodules of unknown origin. She noted fevers, dry cough, chills, and diaphoresis that occurred primarily at night and less frequently during the day. A review of systems was otherwise negative. Her physical exam was unremarkable. She was placed in a negative-pressure room with airborne precautions due to suspicions of tuberculosis, and pulmonology was consulted. Labs on admission revealed new anemia (Hgb 9 g/dL), leukocytosis (WBC 14.0 × 10^9^/L), and thrombocytosis (platelets 543,000/μL) with elevated inflammatory markers and no evidence of eosinophilia. Her renal and liver function panels were unremarkable. Infectious (RSV, Influenza, COVID-19, Fungitell, Aspergillus, cryptococcus, syphilis, and urinalysis) and autoimmune (c-ANCA, RF) workups were negative. An autoimmune lab panel, including ANA and ANCA, was negative. Infectious disease specialists were consulted, and the patient was started on vancomycin, micafungin, and meropenem due to suspicions of septic emboli. The patient underwent a bronchoscopy with bronchoalveolar lavage to evaluate her suspected pulmonary pathology, wherein she developed atrial fibrillation with a rapid ventricular response (RVR) and was started on metoprolol. On biopsy, her tissue was found to be friable with some bleeding noted. During bronchoscopy, the patient went into acute respiratory distress with worsening hypotension refractory to fluids and was started on norepinephrine. She was subsequently intubated and transferred to the intensive care unit (ICU) for treatment of distributive shock and acute hypoxic respiratory failure. Subsequent arterial blood gas analysis during intubation revealed hypoxia with a PaO_2_ of 45, but was otherwise unremarkable. Her MAP was maintained >65 mmHg with norepinephrine and vasopressin, and she remained hemodynamically stable overnight. The patient was extubated and transferred to the medicine floor in stable condition two days later, after being off pressors for more than 24 h. 

The patient remained hemodynamically stable during this time, but her symptoms did not improve, and there was still no evidence of an infectious etiology. The patient’s autoimmune panel revealed the presence of PR3 antibodies, and rheumatology was consulted to evaluate for GPA. The patient underwent a right video-assisted thoracoscopic procedure to resect multiple lung wedges with thoracic surgery for tissue samples and definitive diagnosis. Biopsies of the tissue samples showed necrotizing granulomas and were consistent with the suspected diagnosis of GPA, which was classified as “limited” due to a lack of renal involvement. Her antibiotic prophylaxis was discontinued, and the patient was started on Avacopan and prednisone. Her quantiFERON-TB test was negative, and she was prescribed rituximab inpatient and was continued on this medication outpatient for maintenance therapy. Prior to discharge, she was started on trimethoprim/sulfamethoxazole for Pneumocystis jiroveci pneumonia prophylaxis. The patient’s symptoms, including her fever and sinus pain, subsequently resolved, and she remained hemodynamically stable in the days leading up to her discharge. The patient also began a 7-day course of levofloxacin for Chryseobacterium indologenes, noted on bronchoalveolar lavage culture. She was discharged after a hospital course of three weeks and followed up with rheumatology, infectious disease, cardiothoracic surgery, and her PCP.

## 3. Discussion

In this report, we present the case of a patient with nonspecific upper respiratory symptoms of one month’s duration who visited three different EDs and her PCP twice without a diagnosis or resolution of symptoms. During this time, multiple courses of antibiotics, breathing treatments, and steroids were trialed without relief of symptoms. Numerous studies and imaging modalities were inconclusive leading up to her hospital admission, which was further complicated by her new-onset atrial fibrillation with RVR and hypotension during bronchoscopy. Unlike typical presentations of GPA, this patient originally had a negative ANCA and had no signs or symptoms of kidney injury or involvement with a normal renal function panel [4]. In addition, the patient had no evidence of the typical inciting symptoms of severe GPA, including epistaxis or hemoptysis [2]. 

The differential diagnosis for this patient upon admission included lung malignancy, an infectious process, septic emboli, sarcoidosis, rheumatoid arthritis, GPA, and eosinophilic GPA, given her persistently worsening respiratory symptoms, fever, chills, and night sweats. The reasons for her continuous misdiagnosis at multiple EDs may include her first negative ANCA, her lack of typical GPA presentation, and the timing of her symptoms. Due to the rarity of GPA, physicians may mistakenly believe renal abnormalities to be part of a prototypical diagnostic triad of GPA and thus rule out the pathology in patients with normal renal function. GPA tends to be thought of in this classic generalized presentation, likely because of how GPA is usually taught as well as medical decision-making heuristics. However, presentations of limited GPA do exist without renal involvement, and ANCA positivity can be as low as 60% in these cases [4]. Though 80% of GPA patients eventually develop renal symptoms, less than 20% of individuals have renal involvement at presentation [2]. This case reflects current advances in pharmacologic management of GPA. The combination regimen of rituximab, an anti-CD20 antibody, and avacopan, a C5a receptor inhibitor, offers an effective steroid-sparing approach by reducing complement-mediated neutrophil activation [5,6].

We suggest drawing autoimmune labs, including ANCA, in all patients, especially females, with evidence of lung lesions and unexplained acute respiratory symptoms after ruling out malignancy and infection. Researchers speculate that the X chromosome is involved in the development of autoimmune conditions [7]. Therefore, women (XX) are theorized to have a higher risk of developing autoimmune pathologies as compared to men (XY).

## 4. Conclusions

In conclusion, the diagnosis of GPA should be suspected in all patients with nonspecific constitutional symptoms along with clinical evidence of upper/lower respiratory tract involvement, regardless of renal function. In addition, physicians with a strong suspicion of an autoimmune disease, such as GPA, should utilize a thorough clinical history, physical exam, and other labs in the setting of a negative autoimmune marker and/or negative imaging. Clinical judgment, such as in this case, is required to not rule out GPA despite a negative workup when other more serious causes have been excluded, as the diagnosis may be life-threatening.

## Data Availability

The original contributions presented in this study are included in the article. Further inquiries can be directed to the corresponding authors.
